# Reduced levels of miRNAs 449 and 34 in sperm of mice and men exposed to early life stress

**DOI:** 10.1038/s41398-018-0146-2

**Published:** 2018-05-23

**Authors:** David A. Dickson, Jessica K. Paulus, Virginia Mensah, Janis Lem, Lorena Saavedra-Rodriguez, Adrienne Gentry, Kelly Pagidas, Larry A. Feig

**Affiliations:** 1 Graduate Program in Neuroscience, Sackler School of Graduate Biomedical Sciences, Boston, MA USA; 2 Predictive Analytics and Comparative Effectiveness (PACE) Center, Institute for Clinical Research and Health Policy Studies (ICRHPS), Tufts Medical Center/Tufts University School of Medicine, Boston, MA USA; 3 Division of Reproductive Endocrinology and Infertility, Women and Infants Hospital Fertility Center, Department of OB/GYN, Warren Alpert Medical School of Brown University, Providence, RI USA; 4 The Molecular Cardiology Research Institute, Tufts Medical Center, Boston, MA 02111 USA; 5 Department of Developmental, Molecular and Chemical Biology and Department of Neuroscience, Tufts University School of Medicine, Boston, MA USA; 6 Department of Obstetrics, Gynecology and Women’s Health, University of Louisville School of Medicine, Louisville, KY USA

## Abstract

Exposure of male mice to early life stress alters the levels of specific sperm miRNAs that promote stress-associated behaviors in their offspring. To begin to evaluate whether similar phenomena occur in men, we searched for sperm miRNA changes that occur in both mice and men exposed to early life stressors that have long-lasting effects. For men, we used the Adverse Childhood Experience (ACE) questionnaire. It reveals the degree of abusive and/or dysfunctional family experiences when young, which increases risks of developing future psychological and physical disorders. For male mice, we used adolescent chronic social instability (CSI) stress, which not only enhances sociability defects for >1 year, but also anxiety and defective sociability in female offspring for multiple generations through the male lineage. Here we found a statistically significant inverse correlation between levels of multiple miRNAs of the miR-449/34 family and ACE scores of Caucasian males. Remarkably, we found members of the same sperm miRNA family are also reduced in mice exposed to CSI stress. Thus, future studies should be designed to directly test whether reduced levels of these miRNAs could be used as unbiased indicators of current and/or early life exposure to severe stress. Moreover, after mating stressed male mice, these sperm miRNA reductions persist in both early embryos through at least the morula stage and in sperm of males derived from them, suggesting these miRNA changes contribute to transmission of stress phenotypes across generations. Since offspring of men exposed to early life trauma have elevated risks for psychological disorders, these findings raise the possibility that a portion of this risk may be derived from epigenetic regulation of these sperm miRNAs.

## Introduction

Many studies have confirmed that exposure to severe stress during childhood has long-lasting negative health effects. One of the most convincing has been the Adverse Childhood Experience (ACE) Study, which is supported by over 100 publications^1^. It was initiated by collaboration between the Centers for Disease Control and Prevention and Kaiser Permanente’s Department of Preventive Medicine. It led to the ACE Study Questionnaire (see http://www.acestudy.org/index.html), where anonymous yes or no answers to 10 questions involving participant’s experiences at home until the age of 18 are quantified. Five are personal questions about physical abuse, verbal abuse, sexual abuse, physical neglect, and emotional neglect. Five relate to other family members: an alcoholic parent, a victim of domestic violence, incarceration, diagnosed with a mental illness, and the disappearance of a parent through divorce, death, or abandonment. A score ≥4 puts one at serious risk for future mental and physical health problems, such as a 4.6-fold increased rate of depression^2^ and a ~30-fold increased rate of suicidal ideation and attempts in adults^3^. Remarkably, >10% of the population reports scores of ≥4.

There is a growing appreciation that clinicians should be aware of patients’ traumatic experiences, particularly when young, because they add to their risk for physical and psychiatric maladies^4,5^. Moreover, sensitivity to PTSD has been shown to correlate with ACE score^6–8^ implying it can be used as a screening tool to identify people who should take extra precaution to avoid trauma. However, some may not answer the ACE questionnaire accurately due to suppressed memories or because of the sensitive nature of many of the questions, particularly in settings that do not allow anonymity. Thus, discovery of unbiased markers for early trauma could complement ACE surveys in some clinical settings.

Moreover, offspring of those exposed to early life trauma are at elevated risk for psychiatric disorders^9^. This phenomena has also been demonstrated in rodents^10,11^. For example, transmission of the effects of stress across generations has been observed after exposing male mice to a wide variety of psychological stresses, including social defeat^12^, chronic physical restraint^13^, multiple variable perturbations in adults^14^, social instability beginning in adolescence^15^, and early maternal separation^16^. While some evidence in mice points to environmentally induced changes in sperm DNA methylation as a mechanism for transmission of stress phenotypes^16^, the best evidence to date supports small RNA species in sperm. Recent studies show that sperm contain various types of cytoplasmic RNAs (e.g., mRNAs, miRNAs, siRNAs, lnc-RNAs, piwi-interacting RNAs, and fragments of tRNAs) that have the potential to contribute to embryo development^17–19^. Two sets of reports on psychological stresses in mice implicate miRNAs. In one^20^, early maternal separation of males leads to increased expression of a variety of sperm miRNAs in adult males and depression and social defects in their future offspring. Injection of total sperm RNA from stressed fathers into zygotes mimics some of these effects when these animals matured. In the other^21^, chronic variable stress in mice leads to increases in the expression of nine sperm miRNAs, and when these miRNAs are injected into fertilized zygotes, phenotypes similar to those in offspring from stressed fathers are also observed.

Here, we find that severe early life stress is associated with a reduction in sperm of both mice and men of the levels of multiple members of 34/449 miRNA family that all have the same seed sequence and function together to influence brain development and spermatogenesis^22,23^. miR-34 expression has also been implicated in stress regulation in the adult brain^24–26^. In mice, the effect of stress on these sperm miRNAs crosses generations, as reductions in miR-449a and miR-34c are found in both early embryos derived from stressed fathers and in sperm of males derived from these embryos.

## Materials and methods

### Animals and care facilities

All mice included in this study are of the CD-1 strain, obtained from Charles River Laboratories. Males used for CSI stress began the protocol at 28 days postnatal age, and control females used for breeding were 8 weeks of age. All animals are housed in temperature, humidity, and light-controlled (14 h on/10 h off LD cycle) rooms in a fully staffed dedicated animal core facility led by on-call veterinarians at all hours. Food and water were provided ad libitum. All procedures and protocols involving these mice were conducted in accordance with and approved by the Institutional Animal Care and Use Committee of the Tufts University School of Medicine, Boston, MA. Sample sizes for the mouse work were based upon our previous experience with these experiments in the lab to yield adequate concentration of RNA for downstream work, and no animals were excluded from use in this study. No randomization procedures were needed to separate mice into groups.

### Volunteer recruitment and ACE administration

All research performed on humans and human samples were approved by the University of Louisville School of Medicine Human Subjects Protection Program (IRB #15.1004). We included only Caucasians to limit confounding by race given possible variation in sperm miRNA and response to the ACE questionnaire, and because Caucasians represented the vast majority of patients in the selected source population. Informed consent to participate in this observational study was obtained from all subjects. Eligible men were administered the adverse child experiences (ACE) survey in a self-reporting format after attaining proper consent. Men then provided a semen sample for their initial workup (*n* = 28 samples total). Mature motile sperm were isolated using a Percoll gradient to effectively separate mature sperm from immune cells, dead tissue, immature sperm, and other contaminants^27^. An aliquot of purified sperm was saved for expression analysis, the remainder being used for semen analysis and downstream IVF/ICSI procedures. Demographic and health information for all samples was provided by the clinical team in a de-identified, password-protected spreadsheet to the research team, with a password known only to the authors of this study. All men providing samples for this study were compensated with a $50 gift card. This study was funded to recruit eligible participants for a 1-year period. Given typical clinic volumes and anticipated research participation rates, we expected to recruit a sample of 35 subjects over 12 months. A sample size of 35 subjects achieves 80% power to detect a correlation coefficient of 0.45 for the association between miRNA levels and ACE score using a two-sided hypothesis test with a significance level of 0.05. Recruitment was halted at 28 subjects due to the challenge of lower than expected clinic volume.

### Microarray analysis

Microarray analysis of ten human sperm samples was performed by LC Sciences Inc. (Austin, TX), using 1 μg of total RNA as starting material. Each sample was run on its own custom µParaflo microfluidic chip developed by LC Sciences. Each chip investigates expression of every experimentally verified human miRNA currently listed in miRbase v21. After hybridization, fluorescence images were collected, analyzed, and quantified by LC Sciences to generate a differential miRNA expression analysis, using the appropriate statistical analysis and correcting for multiple comparisons, between our low ACE group (scores 0–1, *n* = 5) and high ACE group (scores >4, *n* = 5).

### RNA extraction and quantitative real-time PCR

Total RNA was extracted from both mouse and human sperm samples using the miRVana miRNA Isolation Kit (Invitrogen) according to the manufacturer's protocol. Total RNA was extracted from mouse embryos using the Norgen Single-Cell RNA Isolation kit (Norgen Biotek Corp.) according to the manufacturer’s protocol. Concentration of RNA was determined using an Agilent 2100 Bioanalyzer (Agilent Genomics) and NanoDrop-1000 (Thermofisher Scientific). Relative gene expression for all samples was determined using the Taqman Advanced cDNA synthesis and qPCR system (Applied Biosystems) purchased from Thermofisher Scientific. An aliquot of 10 ng of total RNA from sperm and 1 ng of total RNA from embryos was used in the initial cDNA synthesis step. Real-time PCR was performed for each target and sample in triplicate on a StepOnePlus PCR System (Applied Biosystems). All data were analyzed using the Comparative ΔΔCT method to generate relative expression data using miR-192-5p as the internal control for all samples.

### Chronic social instability (CSI) stress

Male “F0” juvenile CD-1 mice arrived at P21 and were given a week to acclimate to our mouse facilities. Starting at P28, the composition of each mouse cage (four mice per cage) was randomly shuffled twice per week, for 7 weeks, such that each mouse was housed with three new mice in a fresh, clean cage at each change. This shuffling was randomized to reduce the chance that any mouse would encounter the same mouse twice. Control mice were housed four mice per cage with the same cage mates for the duration of the protocol. After 7 weeks, mice were housed in pairs with a cage mate from the final cage change and left for 2 weeks to remove acute effects of the final change. Male mice (both control and stressed) were then “tease” mated with a naive female mouse for 2 days to increase sperm production. Mice were then either killed for sperm collection or mated with control female mice overnight to generate “F1” animals, with successful mating confirmed via presence of copulation plug the following morning. We have previously shown that stressed males can be mated multiple times and still transmit stress phenotypes to their offspring, so male mice were used for mating, embryo collection, or sperm collection as needed.

### Mouse sperm collection

Mature, motile mouse sperm was isolated via the swim-up method. Briefly, male mice were anesthetized under isoflurane and killed via rapid decapitation. The caudal epididymis and vas deferens were dissected bilaterally and placed in 1 mL of warm (37 °C) M16 medium (Sigma-Aldrich) in a small Petri dish. Under a dissection microscope, sperm were manually expressed from the vas deferens using fine forceps, and the epididymis was cut several times before incubating at 37 °C for 15 min to allow mature sperm to swim out, then large pieces of tissue were removed. The remainder of the extraction took place in a 37 °C warm room. The sperm-containing media was centrifuged at 3000 RPM for 8 min, supernatant was withdrawn and discarded, and 400 µL of fresh, warm M16 medium was then carefully placed on top of the pellet. The tubes were then allowed to rest at a 45° angle for 45 min to allow the motile sperm to swim-up out of the pellet into the fresh medium. The supernatant containing the mature sperm was then carefully withdrawn and combined (*n* = 4–6 mice per group pool) in order to reach sufficient quantities of RNA, and centrifuged again for 8 min at 3000 RPM to pellet the motile sperm. Supernatant was withdrawn and discarded, and the pellet was frozen on dry ice for later processing.

### Mouse embryo collection

To obtain sufficient numbers of embryos, a superovulation protocol was employed. Control adult (8 weeks) female mice were injected with 7.5 U of pregnant mare serum gonadotropin (PMS, National Hormone & Pituitary Program, Harbor-UCLA Medical Center) and 46 h later injected with 7.5 U of human chorionic gonadotropin (HCG, Sigma-Aldrich) and placed into the home cage of the male to mate overnight. In the morning, female mice were checked for copulation plugs and returned to their own cages. If 2-cell and 4-cell embryos were desired, female mice were sacrificed about 1.5 days later, and for 8-cell/morula they were sacrificed about 2 days later. On the day of collection, mice were sacrificed via cervical dislocation. Under a dissection microscope, the uterus was removed and a very high-gauge needle was inserted into the opening of the fallopian tubes and the embryos were flushed out using warm 37 °C EmbryoMax FHM HEPES-buffered medium (1×) w/o Phenol Red (EMD Millipore). The embryos were separated according to cell number, and any unfertilized ova or embryos displaying unusual morphology were discarded. The embryos of interest were pooled by cell stage, and snap frozen on dry ice. Each specific cell-stage sample represents embryos derived from the mating of at least three distinct breeding pairs of mice. Typically, three–four super-ovulated females were mated with three–four males (either F0 control, or F0 stress) for each experiment in order to generate enough embryos needed for a single pool (30–60 embryos total) of a specific cell-stage embryo (2-cell, 4-cell, etc.) to recover sufficient quantities of RNA for downstream analysis. As the females are sacrificed during the collection process, this entire protocol was repeated with new females for every embryo stage collection to generate one pool each of one specific stage embryo for both F0 control fathers and F0 stressed fathers.

### Statistical analysis

Demographic, behavioral, and sperm characteristics were summarized for the study population, stratified by ACE score (low (0–1), medium (2–4), high (≥5)), using the appropriate summary statistic for normally and non-normally distributed data. For all presented data, center values and estimates of variation are stated in the figure legends. For qPCR analysis, miRNA was compared between low (0–1) and high (>4) ACE groups using the Wilcoxon rank-sum test (groups are independent and equivariant). Spearman’s correlation coefficients were used to characterize the strength and direction of the linear relationship between continuous variables. Univariate linear regression analysis was performed to estimate slope coefficients, accompanying *p* values, and 95% confidence intervals. For all analyses described in this section, *p* values are two-sided and alpha was set to 0.05. All statistical analyses were performed in Graphpad Prism v7.0 unless otherwise stated. The nature of this study did not allow for blinding as only objective measurements are reported for these samples.

## Results

### **Inverse association between expression of members of sperm miRNA families 449 and 34 with ACE score of Caucasian males**

To test whether the extent of childhood trauma influences sperm miRNA content in adulthood, 28 Caucasian volunteers, drawn from men giving sperm samples to the University of Louisville Fertility Clinic, filled out the Adverse Childhood Experience (ACE) study questionnaire. The participants were 32.4 years of age on average, with a mean BMI of 26.7 kg/m^2^. Among them, ~20% reported scores ≥4, which is higher than the national average (~12%), but close to the average in Kentucky (~16%) (https://www.childtrends.org/wp-content/uploads/2014/07/Brief-adverse-childhood-experiences_FINAL.pdf). The demographic, behavioral, and sperm characteristics of study participants are summarized in Table 1, stratified by ACE score (low, medium, and high). There were not clinically significant differences noted across the groups defined by ACE score for most parameters. Lower sperm morphology counts were observed in the high ACE group, although a morphology score of 4% is still considered normal according to the most recent World Health Organization guidelines for semen analysis parameters^28^. The other two parameters, sperm count and motility, were also well within normal limits according to these guidelines for all groups (count ≥15 mil/mL; motility ≥40%).

**Table 1:** Demographic, behavioral, and sperm characteristics of the study population, stratified by ACE score

Variable	Low ACE (0–1)	Medium ACE (2–4)	High ACE (≥5)
	*n* = 14	*n* = 7	*n* = 7
Age (years), mean ± SD	30.1 ± 4.1	32.4 ± 6.2	36.7 ± 9.6
BMI (kg/m^2^), mean ± SD	27.1 ± 5.1^a^	26.7 ± 4.8	26.0 ± 3.9^b^
Ever smoked, *n* (%)	8 (57%)	4 (57%)	5 (71%)
Current smoker, *n* (%)	3 (21%)	1 (14%)	2 (29%)
Alcoholic drinks/wk, median (range)	1.5 (0–14)	1 (0–3)	0.25 (0–28)
Illegal drug use, *n* (%)	3 (21%)	3 (43%)	3 (43%)
Sperm count (mil/mL), mean ± SD	84 ± 57	115 ± 62	97 ± 68
Sperm motility (% motile), median (range)	66 (35–96)	72 (44–83)	59 (1–80)^b^
Sperm morphology (% normal), median (range)	8 (2–21)	11 (2–21)	4 (0–12)^b^

^a^*n* = 12 for low ACE group

^b^*n* = 6 for high ACE group

**Fig. 1:**
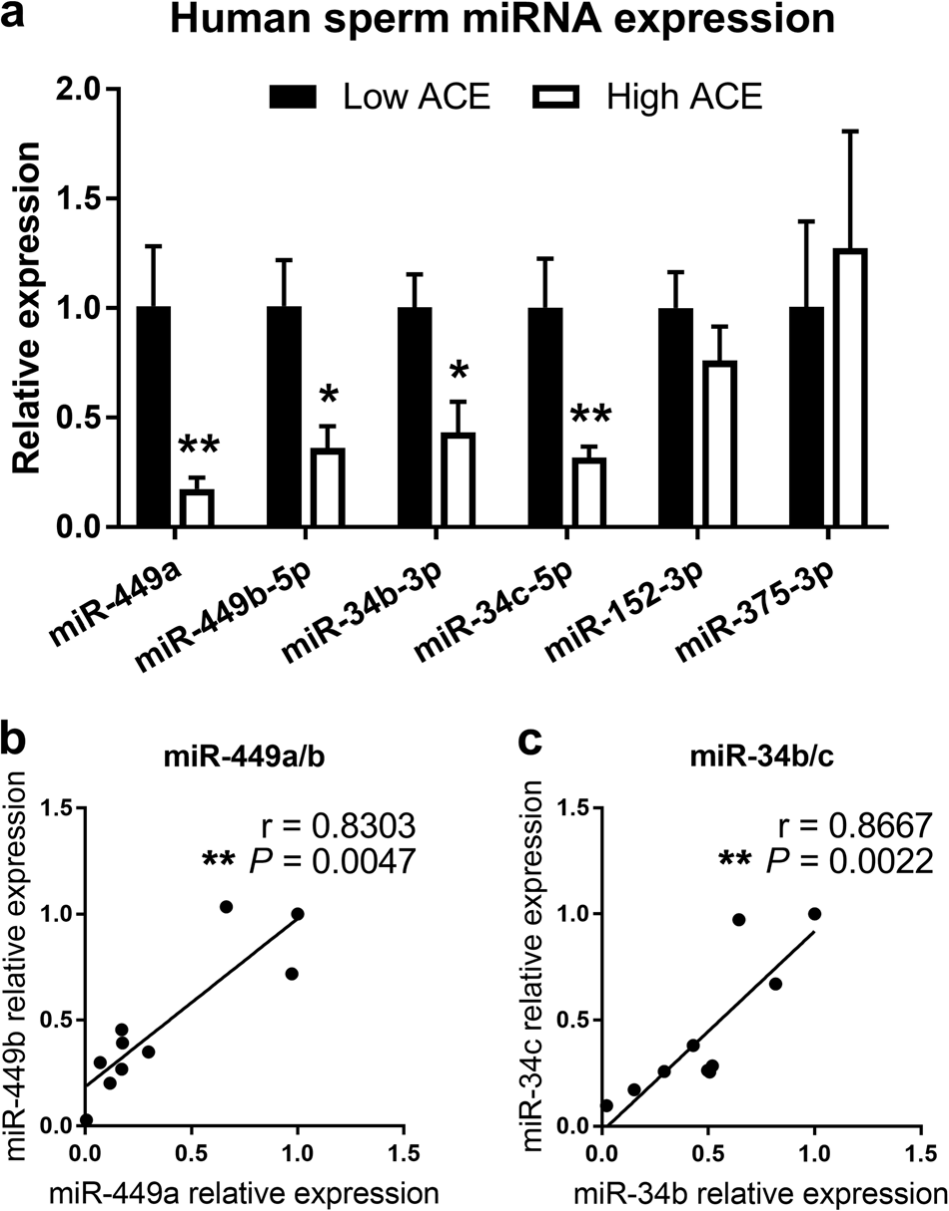
Decreased sperm miRNA expression in men with extensive adverse childhood experiences. **a** qPCR analysis of miR-449a, miR-449b-5p, miR-34b-3p, miR-34c-5p, miR-152-3p, and miR-375-3p in sperm RNA from low ACE group (score 0–1, *n* = 5) vs. high ACE group (score >4, *n* = 5). Wilcoxon rank-sum test *P < 0.05, **P < 0.01. Data represented as mean ± SEM. **b**–**c** Correlation plot comparing relative expression of miR-449a to miR-449b (**b**) and miR-34b to miR-34c (**c**) for individual samples fitted with single-variable linear regression. *n* = 10, *r* = Spearman’s coefficient with exact *p* value listed. Each point represents an individual sample

As a first-level screen to find candidate miRNAs whose expression in sperm correlates with ACE score, we randomly chose sperm samples from five men with the highest ACE scores (≥4) and five with the lowest (0–1) and performed miRNA array analysis on each sample. Among hundreds of miRNAs detected, multiple members of the miRNA family miR-34/449, which all code for the same mRNA inhibitory seed sequence, had the most significant differences in expression between the ACE groups. One set includes two of the three paralogs of miR-449, miR-449a, and miR-449b, and the other set, two of the three paralogs of miR-34, miR-34b, and miR-34c. miR-449c and miR-34a were not detected at reliably quantifiable levels in the arrays. qPCR analysis of these miRNAs revealed an approximately four–fivefold reduction in their levels in sperm samples from the high vs. low ACE score groups (Fig. 1a). To date, no other miRNA tested from this screen has been found to be statistically significantly different between the ACE groups. As examples, we show results for miR-152 and miR-375-3p (Fig. 1a). The former was chosen because array data suggested it might be different, and the latter was chosen because it was identified by two groups as a miRNA elevated in sperm of male mice exposed to two different types of stress^20,21^.

**Fig. 2:**
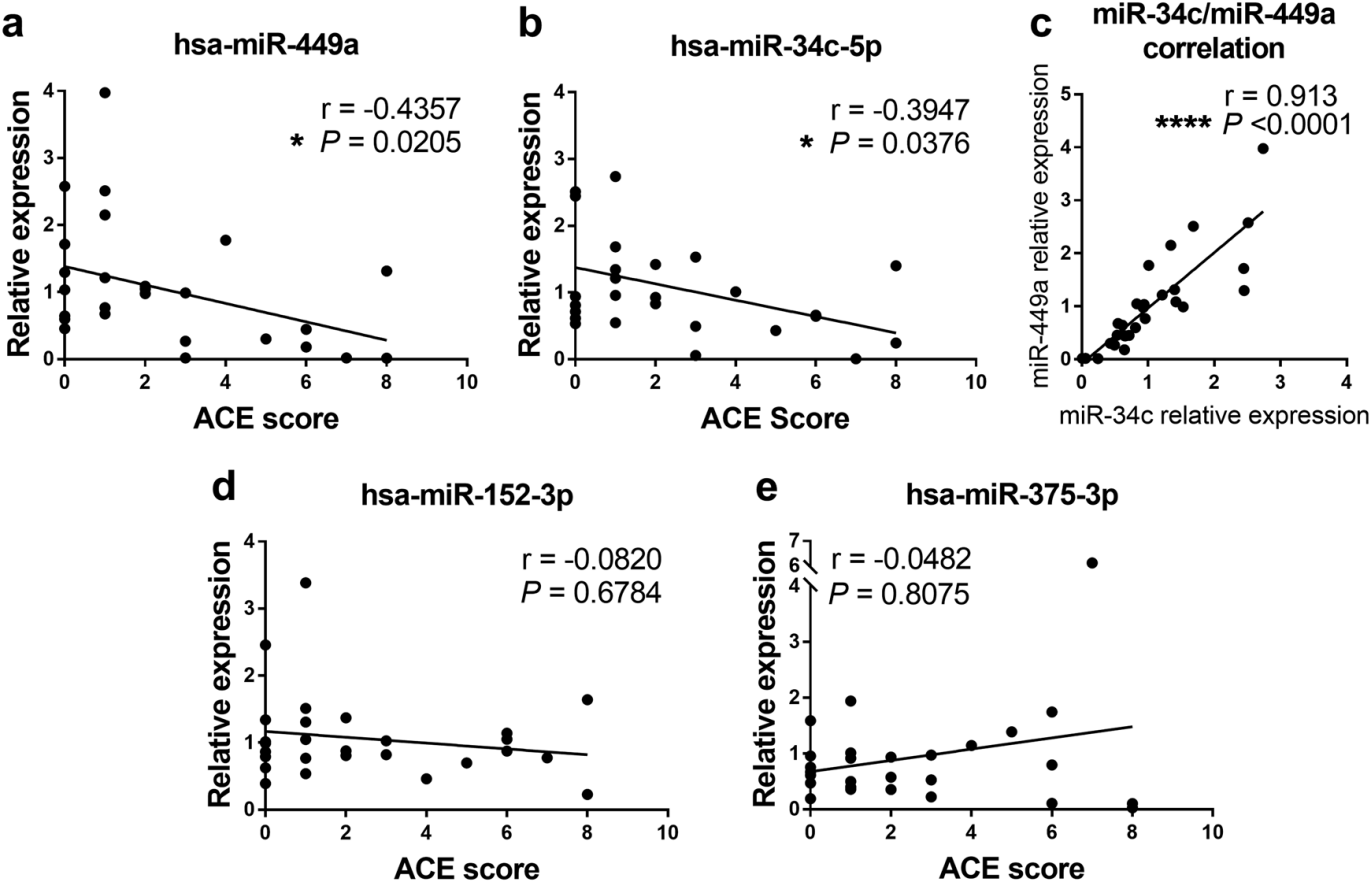
ACE score negatively correlates with expression of miR-449a and miR-34c in 28 human sperm samples. **a**–**b** qPCR analysis of miR-449a, miR-34c-5p, miR-152-3p, and miR-375-3p in all samples, normalized to the overall average expression to generate a relative expression value. Trend lines represent single-variable linear regression. *n* = 28, *r* = Spearman’s coefficient, with *p* values listed. Each point represents an individual sample. **c** Correlation plot comparing miR-449a expression to miR-34c expression in individual samples fitted with single-variable linear regression. *r* = Spearman’s coefficient, with exact *p* value listed. **d–e** qPCR analysis of miR-152-3p and miR-375-3p, data analyzed as in **a**, **b**

We next performed qPCR analysis on these targets in all of the remaining samples. Because the relative levels of miR-449a and miR-449b, as well as miR-34b and miR-34c, were similar to each other in almost every sample (Fig. 1b, c) miR-449a and miR-34c were used as representatives of each family. We found a statistically significant inverse correlation between ACE score and levels of both miRNAs (miR-449a *r* = −0.4357, *P* = 0.0205; miR-34c-5p *r* = −0.397, *P* = 0.0376), where many of the highest ACE score samples had miR-449a and miR-34c levels as much as ~300-fold lower than many of the low ACE score samples (Fig. 2a, b). In contrast, no significant correlations were observed for the association between miR-152 and miR-375-3p and ACE score (Fig. 2d, e). The levels of the miR-449a and miR-34c are coordinately expressed in each sample (*r* = 0.913, *P* = 0.001), implying that stress regulates their levels in sperm by the same mechanism (Fig. 2c).

Sperm miRNA content has been shown to be influenced by smoking^29,30^ and obesity^31,32^; however, in univariate regression analysis neither BMI nor smoking status were significantly associated with expression of sperm miR-449a or miR-34c (Extended Data, Table 1). Moreover, previous studies found that neither smoking nor obesity influence the levels of sperm miR-449a/b or miR-34b/c^30,32^. miRNA expression was also not associated with behavioral characteristics, such as alcohol and drug use, sperm count, or sperm motility. However, a significant association was observed between sperm morphology score and levels of miR-449a, but not miR-34c. Overall, these data imply that early life stress in men, as measured by ACE score, is associated with reduced levels of miR-449a and miR-34c.

### Levels of miR-449a and miR-34c are decreased in sperm of male mice exposed to chronic social instability stress, early embryos derived from them, and sperm from adult mice derived from these embryos

**Fig. 3:**
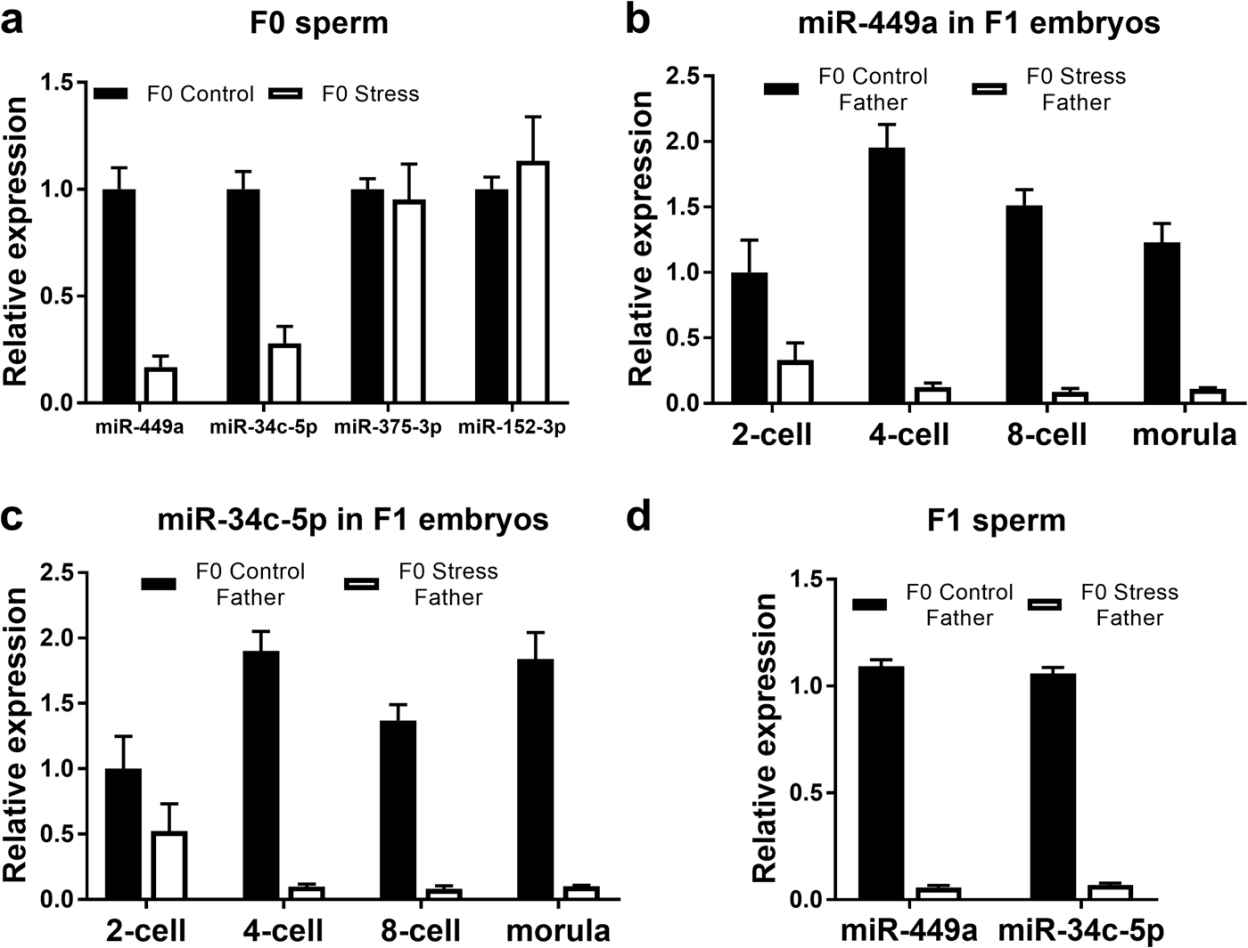
miR-449a and miR-34c are downregulated in sperm across generations and in early embryos derived from them in a mouse model of early life stress. **a** qPCR analysis of miR-449a, miR-34c-5p, miR-152-3p, and miR-375-3p in pooled mature motile sperm isolated from stressed or control mice, *n* = 4–6 males per pool, 1 pool per group. **b**–**c** qPCR analysis of miR-449a (**b**), miR-34c-5p (**c**) in pooled F1 embryos derived from mating control females with stressed or unstressed males. Embryos for each group of fathers and cell stage were pooled prior to analysis (*n* = 30–60 embryos per pool), 1 pool per group. **d** Same analysis as (**a**) performed on sperm from offspring of control and stressed fathers. All samples were pooled before triplicate qPCR analysis, presented as fold change (mean ± experimental error) normalized to **a** F0 control, **b** 2-cell control, **c** 2-cell control, **d** F0 control

To determine whether early life stress also regulates sperm miR-449 and miR-34 in mice, we exposed adolescent males to chronic social instability (CSI) stress^33^, which induces sociability defects in male mice for at least 1 year after stress ceases. It involves changing the cage composition of mice twice a week for 7 weeks beginning during adolescence. We chose this protocol because like adverse childhood experiences, it involves chronic stress when animals are young (adolescents) that leads to long-term negative effects on behavior, it is performed in outbred CD-1 mice that like humans, but unlike inbred mouse strains, are genetically diverse, and because we have replicated its findings^15^. Both miR-449a and miR-34c are sharply reduced in sperm of these stressed males when they are adults (~fivefold) (Fig. 3a). In contrast, and consistent with human results described above, no significant difference was found for sperm miR-152 and miR-375-3p, even though the latter was previously shown to increase after exposure of male mice to two different stress paradigms^20,21^.

Recently, we^15^ and then others^13^ showed that CSI stress has transgenerational effects through the male lineage that induces anxiety and sociability defects specifically in F1 female offspring of stressed males. Moreover, even though the F1 males showed no anxiety or sociability defects, we showed that they induce both behavioral defects specifically in their F2 female offspring^15^. A distinguishing feature of miR-449a/b and miR-34b/c is that they are among a small set (14) of miRNAs in mice that are not present in oocytes but delivered to them from sperm upon fertilization^34^, suggesting that changes in these miRNAs observed in sperm of stressed males may influence subsequent generations via alterations in early development. Thus, 2 weeks after cessation of CSI stress exposure, stressed male mice and control counterparts were mated and early embryos were collected. Consistent with this hypothesis, we discovered large decreases in the expression of both miR-449a and miR-34c in embryos isolated at the 2-cell, 4-cell, 8-cell, and morula stages (~two–tenfold) from the mating of stressed males to control females, compared to those from the mating of control males (Fig. 3b, c).

When male embryos from stressed fathers reached adulthood, levels of their sperm miR-449a and miR-34c were assayed, and found to also be severely suppressed (~tenfold) compared to those from control males (Fig. 3d), consistent with our previous finding that these mice transmit stress phenotypes to their F2 offspring^15^.

## Discussion

In this study of Caucasian men, an inverse association was found between a measure of exposure to abusive and/or dysfunctional family behavior and sperm levels of multiple members of the miR-449/34 family. Prior studies in humans have documented environmental effects on sperm RNAs. However this study is the first to show that stress affects the levels of human sperm miRNAs, and those found reduced are different from miRNAs previously detected in response to smoking^30^ or obesity^32^. In addition, the ACE questionnaire used in this study is designed to capture stressful events occurring ~20 years earlier during one’s childhood. Moreover, although a high ACE score (>4) implies a very substantial health risk, only a minority of men (~20–30%) in this category present with significant psychological and physical problems as adults^1^. Despite this, almost all of the men with high ACE scores in our study displayed reduced levels of these miRNAs. If this pattern holds in a larger independent cohort of men, it would indicate that high levels of maltreatment and household dysfunction in childhood are associated with altered sperm miRNA profiles in adulthood regardless of whether men are susceptible or resistant to the psychological burdens associated with high ACE scores.

This is not to imply that current psychiatric state or recent traumatic experiences do not also suppress these sperm miRNAs. In fact, experiments reported here in mice detected severely reduced levels of sperm miRNAs 449a and 34c only 2 weeks after the cessation of social instability stress. Thus, the low levels of sperm miRNAs 449a and 34c observed in some men in this study with low ACE scores could be a consequence of their present psychiatric state or exposure to recent severe stressful experiences. They could also be due to early life traumas that are not surveyed in the ACE questionnaire, such as being bullied. In addition, low-sperm miRNA expression in men reporting low ACE scores could be due to the fact that some people under-report their adverse childhood experiences^35^. This could be because the survey asks sensitive questions such as exposure to sexual abuse that many are uncomfortable admitting occurs, or because some have suppressed these memories. These possibilities support a larger follow-up study, now in the planning stage, that surveys men’s present psychiatric state along with their ACE scores and sperm miRNA levels. This may reveal that low levels of sperm miRNAs 449a and 34c are associated with men’s present psychiatric problems, in addition to their early exposure to abuse, which could have translational significance.

Interestingly, both sharply reduced levels of sperm miR-449 and miR-34 family members and severe stress have been found to be associated with reduced sperm quality and fertility in men^32,36^. Consistent with these observations, we found low-sperm morphology score associated with low miR-449a expression. Additionally, sperm count and sperm motility showed a trend toward associating with expression of both miRNAs, although these were not significant. Thus, we hypothesize that the connection found previously between stress and sperm quality and fertility may be through, at least in part, stress effects on the levels of sperm miRNAs 449a/b and 34b/c.

This study is the first to identify novel sperm miRNA changes associated with exposure to childhood trauma in men. The relatively small sample size limits our ability to more deeply explore the association between ACE scores and miRNA expression, including additional mediating variables. Nevertheless, these findings will serve to inform a subsequent larger study that can support control for additional confounding factors in a multivariable environment. In addition, this was a cross-sectional design with only a single measurement of miRNA and assessment of exposure to childhood trauma. Longitudinal studies with information on behavioral and psychological factors through adulthood, with repeated measurements of sperm miRNA content, could illuminate how cumulative exposures affect miRNA levels, potential mediating factors and the durability of these effects and the association between these sperm miRNAs levels and fertility in men.

The discovery that low levels of multiple members of the miR-34/449 gene family are associated with men’s high ACE scores also has potential implications for the next generation. A growing body of evidence, including phenotypes of knockout mice that cannot process miRNAs^34^, supports the idea that paternal miRNAs are delivered to the zygote after fertilization and contribute to early embryo development. Also, other stress paradigms in male mice have been shown to increase expression of specific sperm miRNAs (not miRs 449 and 34), and injection of them into zygotes fertilized by normal males induces a stress phenotype in offspring that is consistent with that obtained by mating stressed fathers^20,21^. Interestingly, among identified sperm miRNAs, only miR-449a/b and miR-34b/c have been shown to be expressed specifically in sperm, not eggs, and are present in zygotes, supporting a mechanism for how the small amounts of these miRNAs in sperm can impact early development. Moreover, in humans, sperm miR-34b/c levels correlate with day-3 embryo quality, implantation rate, and pregnancy after intra-cytoplasmic sperm injection (ICSI) procedures used in fertility treatment, implying this sperm derived miRNA influences early development in humans^37^. Finally, the present study is unique among studies implicating sperm miRNAs in the transgenerational effects of stress by demonstrating that the same stress-induced miRNA expression level changes that occur in sperm of stressed male mice, persist in F1 embryos derived from them, and in sperm of F1 male mice derived from these embryos, which we showed previously^15^ pass on stress-associated traits to their F2 offspring.

If a similar phenomenon occurs in humans, we predict that reductions in the levels of sperm miR-449a and miR-34c we detect in men with high ACE scores could be transmitted to their sons. If so, this process could yield another explanation for why some men in our survey display low miR-449a and miR-34c levels despite reporting low ACE scores. They may have inherited these sperm changes from their fathers because of their exposures to abusive and/or dysfunctional family life, a possibility we are planning to test in a follow-up study determining whether fathers of these men have higher than average ACE scores.

miR-449 and miR-34 have the same inhibitory seed sequence and function together in mouse development, such that knockout of either miR-449 or miR-34 paralogs alone does not yield a detectable developmental phenotypes, whereas knockout of both sets of miRNAs mice show defects in brain development and spermatogenesis caused, at least in part, by defective microtubule and associated cilia function^22^. The fact that we detect reduction in expression of paralogs of both miR-34 and miR-449 genes in sperm of men with high ACE scores, as well as in sperm of mice exposed to sociability stress and in embryos derived from them, adds to the potential functional significance of these findings.

The gene targets of these miRNAs that carry out the described developmental functions in both mice and humans have not been clarified, but documented targets in other cell types where these miRNAs are also expressed include p53, CDK6, c-MYC, HDAC1, and BCL-2, all of which could have long-lasting effects on embryo development^38^. Thus, the severe decrease in expression of both miR-449a and miR-34c we detect in early embryos from stressed male mice could alter brain development and the process of spermatogenesis in subtler ways than knockout mice. These developmental changes may contribute to the enhanced anxiety and defective sociability we observed in female offspring, and altered sperm miRNAs we detect in male offspring of male mice exposed to chronic social instability stress^15^, a question now being addressed.

Interestingly, miR-34 paralogs have also been implicated in regulating the stress response in adult mice, although the findings are somewhat contradictory^24–26^. However, our evidence to date suggests those effects may be distinct from the transgenerational effect we have observed. First, the effects observed in adults have been detected in male mice, whereas we detect stress-related phenotypes only in adult female offspring of stressed males. Second, complete knockout of all miR-34 paralogs has no effect on basal anxiety, only changes induced by acute stress^22^. In contrast, we detect basal changes in anxiety and sociability in female offspring of stressed males. Third, our phenotype is likely the consequence of suppressing both 34 and 449 members of the miRNA family. Finally, we have observed severe decreases in miR-34c levels in early embryos derived from stressed males. However, to date we have not been able to detect significant changes in its levels in brain regions of adult females derived from these embryos, where these studies have documented the consequences of altering all three paralogs of miR-34.

It is known that children of parents who report high ACE scores are at higher risk for stress-associated behavioral problems, which has been assumed to be due to parental behaviors^39,40^. This study raises the possibility that another component is stress-induced miRNA changes in men’s sperm. Thus, just as genetic testing of sperm DNA can assess risk across generations, future studies based on these findings may reveal value in epigenetic testing of sperm miRNA as well.

### Data availability

Data are available upon reasonable request.

## Electronic supplementary material

Extended Data
