# Supplementary material for: Reduced levels of miRNAs 449 and 34 in sperm of mice and men exposed to early life stress
Source: Transl Psychiatry. 2018 May 23;8:101. doi: 10.1038/s41398-018-0146-2 (PMC5966454; doi:10.1038/s41398-018-0146-2)
Supplement: Supplementary file 1 — Extended Data [file 41398_2018_146_MOESM1_ESM.docx]

Extended Data Table 1 | Univariate linear regression analysis of behavioral and sperm characteristics association with sperm miRNA levels

|  | miR-449a | | | | miR-34c | | | |
| --- | --- | --- | --- | --- | --- | --- | --- | --- |
| Variable | Beta | 95% lo | 95% hi | *p*-value | Beta | 95% lo | 95% hi | *p*-value |
| BMI | -0.0374 | -0.1230 | 0.0483 | 0.376 | -0.0374 | -0.1084 | 0.0336 | 0.287 |
| Smoking | -0.1587 | -0.6180 | 0.3007 | 0.484 | -0.1697 | -0.5281 | 0.1887 | 0.340 |
| Drug use | -0.3066 | -1.0500 | 0.4363 | 0.404 | -0.1191 | -0.7097 | 0.4715 | 0.682 |
| Alcohol use | -0.4969 | -1.2840 | 0.2903 | 0.206 | -0.2419 | -0.8735 | 0.3897 | 0.438 |
| Sperm count | 0.0036 | -0.0022 | 0.0094 | 0.212 | 0.0026 | -0.0020 | 0.0072 | 0.264 |
| Sperm motility | 0.0124 | -0.0042 | 0.0290 | 0.135 | 0.0098 | -0.0030 | 0.0226 | 0.127 |
| Sperm morphology | 0.0583 | 0.0020 | 0.1146 | **0.043** | 0.0237 | -0.0226 | 0.0701 | 0.302 |

Regression analysis performed on all samples with known values of all variables of interest (n=25) using Graphpad Prism v7.0 for all parameters listed. For smoking and Alcohol, use was quantified as an ordinal variable with no smoking/drinking=0, past smoking drinking=1, current smoking/drinking=2, >1 drink/day=3. Significance considered p<0.05. BMI, Body Mass Index (kg/m2).
